# Gross hematuria as the presentation of an inguinoscrotal hernia: a case report

**DOI:** 10.1186/1752-1947-5-561

**Published:** 2011-12-04

**Authors:** Kayvan Ansari, Mohammad Reza Keramati, Kiara Rezaei Kalantari, Maryam Jafari, Gholamali Godazandeh, Mohsen Pakzad

**Affiliations:** 1 Department of General Surgery, Firoozgar Hospital, Tehran University of Medical Sciences, Tehran, Iran; 2 Department of Radiology, Firoozgar Hospital, Tehran University of Medical Sciences, Tehran, Iran; 3 Department of General Surgery, Mazandaran University of Medical Sciences, Sari, Iran; 4 Department of General Surgery, Arak University of Medical Sciences, Arak, Iran

## Abstract

**Introduction:**

Several complications have been reported with inguinal hernias. Although hematuria and flank pain, either as the presentation or as a complication of inguinal hernia, are infrequent, this condition may lead to the development of obstructive uropathy, which can have diverse manifestations.

**Case presentation:**

A 71-year-old Iranian man with Persian ethnicity presented with new onset episodes of gross hematuria and left-sided flank pain. A physical examination revealed a large and non-tender inguinal hernia on his left side. An initial workup included an abdominal ultrasound, an intravenous pyelogram and cystoscopy, which showed left hydronephrosis and a bulging on the left-side of his bladder wall. On further evaluation, computed tomography confirmed that his sigmoid colon was the source of the pressure effect on his bladder, resulting in hydroureteronephrosis and hematuria. No tumoral lesion was evident. Herniorrhaphy led to the resolution of his signs and symptoms.

**Conclusion:**

Our case illustrates a rare presentation of inguinal hernia responsible for gross hematuria and unilateral hydronephrosis. Urologic signs and symptoms can be caused by the content of inguinal hernias. They can also present as complications of inguinal hernias.

## Introduction

Hematuria may reflect either significant nephrological or urological disease. Hematuria of nephrological origin is frequently associated with casts in the urine and almost always associated with significant proteinuria [1]. Isolated hematuria without proteinuria, other cells or casts is often indicative of bleeding from the urinary tract. Hematuria is defined as two to five red blood cells (RBCs) per high-power field (HPF). Common causes of isolated hematuria include stones, neoplasms, tuberculosis, trauma and prostatitis. Gross hematuria with blood clots almost never has a glomerular basis;rather, it suggests a postrenal source in the urinary collecting system. The likelihood of urogenital neoplasms in patients with isolated painless hematuria (nondysmorphic RBCs) increases with age. Hematuria with pyuria and bacteriuria is a typical presentation for infection. Acute cystitis or urethritis in women can cause gross hematuria. Hypercalciuria and hyperuricosuria are also risk factors for unexplained isolated hematuria in both children and adults [2]. Herein, we present a patient with a left inguinal hernia resulted in flank pain, hydronephrosis and hematuria.

## Case presentation

A 71-year-old Iranian man with Persian ethnicity presented to our urology clinic complaining of recurrent episodes of gross hematuria and left-sided flank pain of one week's duration. He mentioned no history of trauma, prior disease, medication usage or significant family disorder. On physical examination, he was fully conscious with a blood pressure of 110/70 mmHg, pulse rate of 120 beats/min and temperature of 37°C. His bowel sounds were normoactive on auscultation. There were no remarkable findings on abdominal palpation, except for a non-tender, large left inguinal hernia with extension to his scrotum. Other related examinations, including a digital rectal examination, were normal. A urine analysis showed pH 5, with 10 to 15 RBCs per HPF, 10 white blood cells per HPF, with rare bacteria and yeast. A urine culture was negative.

**Figure 1:**
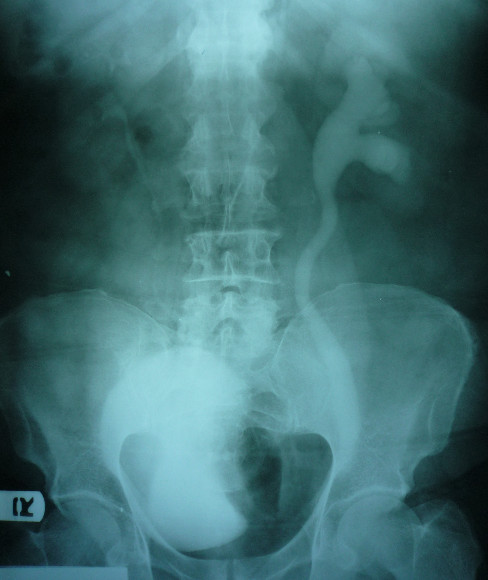
**Preoperative intravenous pyelogram showing left hydroureteronephrosis**.

**Figure 2:**
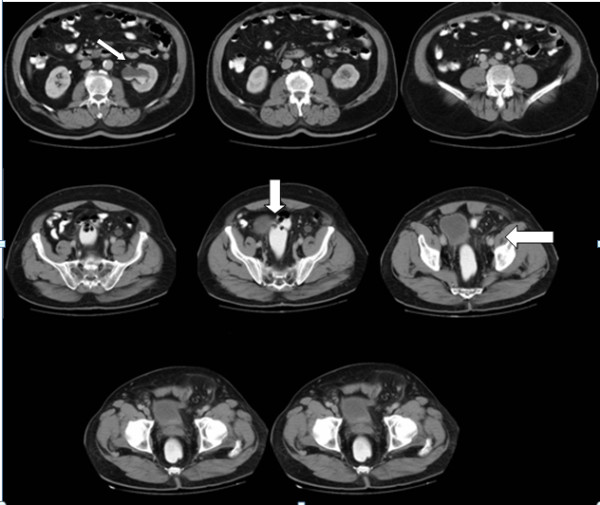
**Preoperative abdominopelvic computed tomography scan.** Left hydroureteronephrosis (thin arrow). Distal ureteral obstruction and bladder displaced by entrapped herniated sigmoid colon (thick arrow).

Genitourinary ultrasonography reported a grade 2 hydroureteronephrosis on his left side. An intravenous pyelogram also revealed left hydroureteronephrosis associated with an ill-defined filling defect on the left side of his urinary bladder (Figure 1). In order to rule out any intravesical lesion, a cystoscopy was performed that showed a bulging on the left side of his bladder wall due to extravesical pressure. The mucosal lining of his bladder was normal. Intravenous and oral contrast-enhanced abdominal computed tomography (spiral multislice thin section scan) showed an entrapped sigmoid colon that was herniated through his left inguinal canal. Anteromedial displacement of his bladder and left ureter were also evident due to the pressure of his sigmoid colon. His left ureter was dilated due to distal obstruction (Figure 2).

A diagnosis was made of a large inguinal hernia with pressure effects on the urinary system, resulting in hematuria and obstructive hydroureteronephrosis;the abdominal wall was thus opened with a classic inguinal incision. The contents of his hernial sac, including his sigmoid colon and its mesentery, were adhered to the surrounding tissues. An attempt to reduce the content of his hernial sac was unsuccessful, so a low midline incision was made for better exposure and reduction. There was no intra-abdominal mass. A redundant sigmoid colon was found fixed at the internal ring due to severe and chronic adhesion. His proximal sigmoid colon had compressed his bladder and distal ureter at the vesicoureteral junction. After reduction of his hernial sac content, our patient underwent a successful hernia repair with mesh, leading to a quick and uneventful postoperative recovery.

**Figure 3:**
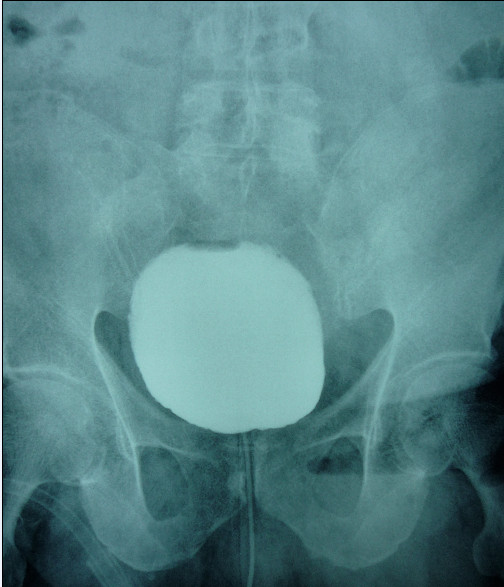
**Postoperative cystogram showing reversal of all prior radiological signs**.

Our patient's signs and symptoms subsided following surgery. On a postoperative cystogram, all signs had disappeared (Figure 3). Our patient was initially followed-up with monthly visits for the next six months, and then every six months. He remained symptom free during postoperative follow-up.

## Discussion

Although hematuria presents with many urologic diseases, the acute onset of gross hematuria is almost always indicative of a postrenal pathology. This includes stones, infection, trauma and tumor.

Inguinal hernias present along a spectrum of scenarios. These range from incidental findings to surgical emergencies such as incarceration and strangulation of the hernia sac contents. Asymptomatic inguinal hernias are frequently diagnosed incidentally on physical examination or may be brought to the patient's attention as an abnormal pain-free bulge. In addition, these hernias can be identified intra-abdominally during laparoscopy [3]. Patients who present with a symptomatic inguinal hernia will frequently present with groin pain. Less commonly, patients will present with extra-inguinal symptoms such as a change in bowel habits or urinary symptoms. Regardless of size, an inguinal hernia may impart pressure onto proximal nerves, leading to a range of symptoms. These include generalized pressure, local sharp pains and referred pain. Lastly, neurogenic pains may be referred to the scrotum, testicle or inner thigh. A change in bowel habits or urinary symptoms may indicate a sliding hernia consisting of intestinal contents or involvement of the bladder within the hernia sac [3].

The presence of urological symptoms and signs such as hematuria, flank pain and hydroureteronephrosis may be seen with an inguinal hernia, but they generally occur when there is associated bladder herniation [4-6]. A broad literature search revealed that the presence of the bladder within an inguinal hernia occurs in approximately 1% to 4% of all adult hernia cases. Ureteroinguinal herniation was also reported and seems to be an even more rare entity [7].

Moreover, although urological symptoms and signs are often the predominant clinical symptoms when herniation of urinary organs occurs, our case revealed that urological features could be present even in the absence of urinary organ herniation. We think that, given the resultant urinary retention, high intraluminal pressure and consequent urothelial injury in the ureter and upper urinary system, the hematuria occurred as a rare presentation of an inguinal hernia. So, in this clinical pattern, care must be taken to prevent probable morbidities through either diagnostic or therapeutic procedures.

Ruling out urinary organ herniation during preoperative work-up is helpful to avoid surgical complications, the most common being ureteral injury. Ultrasound may be helpful in diagnosis, with its ability to demonstrate hydronephrosis and, occasionally, the herniated urethra in the inguinoscrotal region, but it has limited value in showing the herniated ureter and a high index of suspicion is required. Nevertheless, in this case, ultrasound could be the first step in diagnostic imaging since urinary symptoms were present. Intravenous pyelography may aid the diagnosis by showing an intact non-herniated ureter. Computed tomography is the preferred imaging modality with high spatial resolution, not only to detect herniation, but also to show associated pathologies.

## Conclusion

Our case illustrates a rare presentation of inguinal hernia, responsible for gross hematuria and unilateral hydroureteronephrosis. Urologic signs and symptoms can be induced by the contents of an inguinal hernia. They can also be present as complications of an inguinal hernia.

## Consent

Written informed consent was obtained from the patient for publication of this case report and any accompanying images. A copy of the written consent is available for Review by the Editor-in-Chief of this journal.

## Competing interests

The authors declare that they have no competing interests.

## Authors' contributions

Authors were all involved in the drafting and re-editing of the manuscript. The final manuscript was read and approved by all six authors.
